# Significant increase in calcification and ossification observed after the second relapse of low-grade myofibroblast sarcoma treated with radiotherapy followed by anlotinib and toripalimab: a case report

**DOI:** 10.3389/fimmu.2025.1606825

**Published:** 2025-11-27

**Authors:** Juan Lang, Zhongkui Xiong

**Affiliations:** 1 Department of Pathology, Shaoxing People’s Hospital, Shaoxing, Zhejiang, China; 2 School of Medicine, Shaoxing University, Shaoxing, Zhejiang, China; 3 Department of Radiation Oncology, Shaoxing Second Hospital, Shaoxing, Zhejiang, China

**Keywords:** myofibroblast sarcoma, radiotherapy, immunotherapy, calcinosis, toripalimab, anlotinib, drug therapy

## Abstract

**Introduction:**

Low-grade myofibroblastic sarcoma (LGMS) is a rare malignant neoplasm of the soft tissues, originating from stromal cells and accounting for approximately 1% of all malignant soft tissue tumors. It is characterized by its invasive nature, high rates of recurrence, and the presence of calcification. To date, no established treatment strategy exists for relapsed LGMS. This study aims to provide a case treated with combination regimen of palliative radiotherapy followed by toripalimab and anlotinib for LGMS patients with the second relapse, to evaluate its efficacy and safety, and to explore the impact of tumor calcification and ossification on subsequent treatment outcomes and prognosis.

**Methods:**

This report presents the case of a 23-year-old male LGMS patient with two relapses who initially presented with post-tracheotomy status due to pharyngeal obstruction caused by masses located at the base of the tongue and floor of the mouth that had persisted for 3 weeks. He underwent palliative radiotherapy followed by toripalimab combined with anlotinib. Subsequently, surgical resection of residual lesions performed.

**Results:**

CT imaging demonstrated a significant increase in calcification and ossification of the second relapsed lesions and lymph nodes in the draining region, and stable disease with tumor shrinkage to these interventions of palliative radiotherapy followed by toripalimab combined with anlotinib. The patient was followed up until February 2025, achieving a progression-free survival 2 of 48 months from initiation of radiotherapy and an overall survival of 9 years.

**Discussion:**

Based on this case report, palliative radiotherapy followed by anlotinib and toripalimab may yield an acceptable therapeutic effect while being associated with manageable treatment-related adverse events in LGMS patients experiencing two relapses, who have previously undergone targeted therapy using vascular endothelial growth factor receptor-tyrosine kinase inhibitors and have not received prior radiotherapy. Nevertheless, this hypothesis requires further validation through higher-level evidence-based medical research.

## 1 Introduction

Low-grade myofibroblastic sarcoma (LGMS) is a malignant neoplasm of the soft tissues that arises from stromal cells (1). LGMS is a rare and relatively recent classification (2) that was first recognized and described by Mentzel et al. in 1998 (3). In 2002, the World Health Organization incorporated this tumor into the International Classification of Diseases for Oncology, which remained in effect until 2020 (4). The classification designates it solely as LGMS, but the definitions of intermediate-grade myofibroblastic sarcoma and high-grade myofibroblastic sarcoma remain controversial (5). LGMS is most frequently found in extremities as well as the head and neck region, particularly within the oral cavity—especially on the tongue, mandible, and larynx (6, 7). LGMS is an atypical tumor characterized by the presence of myofibroblasts exhibiting a variety of histological patterns, exhibiting features reminiscent of fibromatosis (1). LGMS is distinguished by the presence of calcification. Wang et al. reported that 2 out of 14 cases demonstrated significant calcification and ossification (8). LGMS accounts for approximately 1% of all malignant soft tissue sarcomas (STSs) (9) and predominantly affects middle-aged men (9, 10). Data from the Surveillance, Epidemiology, and End Results (SEER) database for 96 patients diagnosed with LGMS indicate that the mean overall survival (OS) was 125.2 months. The OS rates at 1, 3, 5, and 10 years were reported to be 88%, 77%, 70%, and 59%, respectively (11).

A population-based study involving 49 cases suggests that surgical intervention is the most common treatment approach (12). Surgical resection continues to be recognized as the most effective therapeutic strategy for LGMS (11). Furthermore, a systematic review encompassing 43 studies and 78 cases has confirmed that complete surgical excision remains the preferred treatment option (13). According to a multicenter study conducted by the Japanese Musculoskeletal Oncology Group, wide excision aimed at achieving microscopically negative (R0) margins is considered the standard treatment for LGMS (14). Therefore, wide excision with R0 margins should be regarded as the standard of care for LGMS (15). Nevertheless, the optimal surgical extent remains to be further elucidated. Notably, this neoplasm is associated with a high risk of local recurrence, yet exhibits a relatively low risk of distant metastasis (16). Radiotherapy (RT) may serve as a viable alternative in cases where surgical resection is not feasible or when surgical intervention is expected to lead to significant functional impairment (14). Additionally, adjuvant RT or chemoradiotherapy can be considered (17). On the contrary, Mamikunian et al. reported that adjuvant RT did not show any significant improvement in recurrence rates (18). The role of adjuvant RT in these patients remains ambiguous (18) and necessitates further investigation (12).Chemotherapy has demonstrated limited efficacy in improving survival outcomes (11). Therefore, it is recommended that chemotherapy should not be routinely administered in cases of LGMS, particularly for patients who present with negative surgical margins postoperatively (11).

Despite their relatively low malignant potential, these tumors are highly invasive, tend to recur, and may metastasize to distant sites (17). Long-term outcomes of LGMS, as observed in a single-center case series involving 15 patients, indicate that local tissue invasion and the surgical approach employed may be correlated with the incidence of local recurrence (19). However, owing to its rarity, the precise biology of this tumor is poorly understood, and its treatment protocols and prognosis remain suboptimal (17). To date, no treatment strategy has been established for recurrent LGMS (7). Herein, we present a case of a 23-year-old male diagnosed with the second relapse of LGMS who exhibited stable disease with a tendency toward tumor reduction following treatment with palliative RT followed by anlotinib combined with toripalimab.

## 2 Case presentation

**Figure 1:**
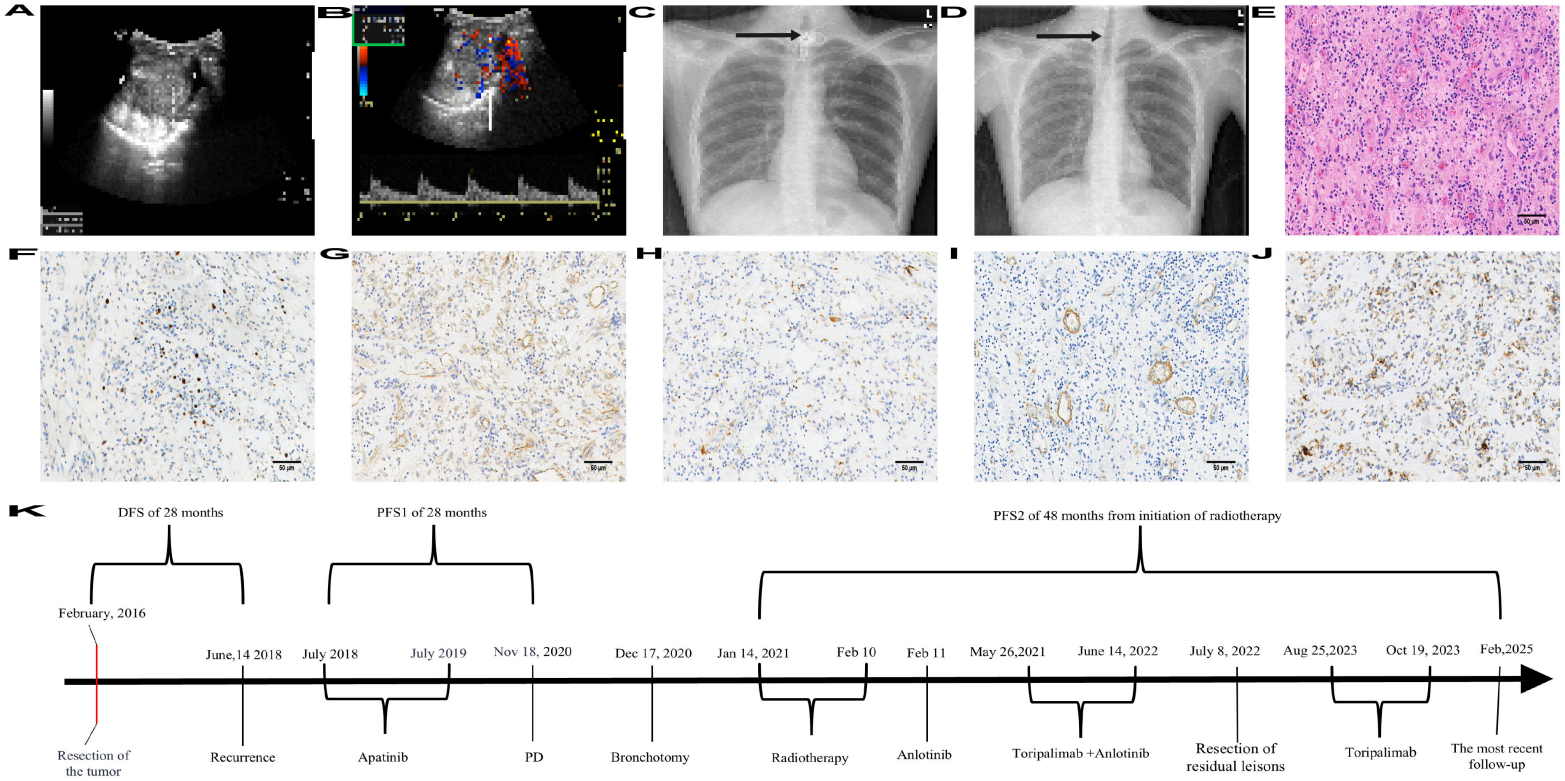
Imaging and pathological examination of the patients. **(A, B)** Ultrasound examination images conducted on June 14, 2018. **(A)** A hypoechoic mass measuring 53 × 47 × 55 mm was identified, extending from the base of the tongue to the floor of the mouth. The lesion exhibited a well-defined boundary and an irregular shape. **(B)** Intralesional blood flow signals are abundant, as demonstrated by imaging analysis. **(C, D)** Chest radiograph. **(C)** The arrow denotes the tracheotomy tube, which was present prior to the surgical resection of the residual lesion, as evidenced by an X-ray image on April 27, 2021. **(D)** The arrow denotes a location on the chest radiograph, dated November 28, 2022, where the tracheotomy tube was absent, which had been removed simultaneously following the surgical resection of the residual lesion. **(E-J)** Pathological examination images and immunohistochemical analysis results. The magnification was set at 200 ×, and the scale bar represented 50 microns. **(E)** The hematoxylin and eosin (H&E) stained image. **(F-J)** Images of immunohistochemical analysis results. **(F)** Ki 67 (+, 5%). **(G)** β-Catenin demonstrated plasma positivity in a small fraction. **(H)** Desmin, a limited degree of weak positivity. **(I)** SMA, negative. **(J)** PD-L1, CPS: approximately 30, as determined using the 22C3 assay. **(K)** The timeline of the course detailing the patient’s treatment. After the first recurrence, the patient underwent anti-VEGFR targeted therapy with apatinib mesylate tablets but discontinued due to intolerance of adverse reactions. Following the second relapse, he underwent RT followed by anti-VEGFR targeted therapy with anlotinib capsules combined with toripalimab-based immunotherapy. The disease-free survival (DFS) is reported to be 28 months, while the progression-free survival at first assessment (PFS1) also stands at 28 months. Additionally, a PFS2 of 48 months has been observed following the initiation of radiotherapy (RT), with an overall survival (OS) extending to 9 years.

**Figure 2:**
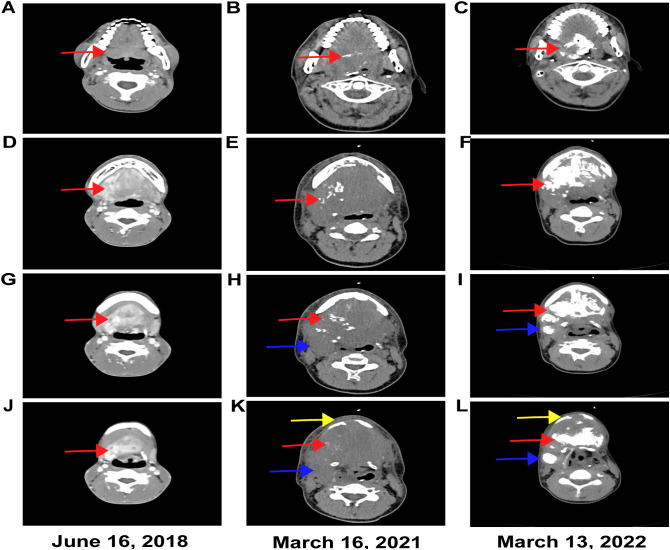
As demonstrated in the CT images labeled **(A, D, G, J)**, which were captured on June 16, 2018. A clump-like soft tissue density shadow was identified in the right mandibular angle region. Heterogeneous enhancement was observed on the enhanced scan images. This marks the first recurrence of the tumor. There was an absence of calcification in both the tumor region and the lymph node drainage area. As showed in the CT images labeled **(B, E, H, K)**, which were conducted on March 16, 2021. In the right mandibular angle region, an irregular soft tissue density mass measuring approximately 88 × 79 × 65 mm was identified. This mass demonstrated multiple punctate calcifications and was associated with destructive changes in the adjacent bone. This moment occurred after the completion of palliative radiotherapy and prior to the initiation of treatment with anlotinib in combination with toripalimab for the second relapse. As showed in the CT images labeled **(C, F, I, L)**, which were conducted on March 13, 2022. A mass measuring up to 84 mm in maximum diameter was identified in the right mandibular angle, associated with significant calcification and adjacent bone destruction. The red arrow indicated tumor extension from the base of the tongue to the floor of the mouth. The yellow arrow highlighted mandibular destruction, and the blue arrow denoted lymph nodes located in the Ib region.

The young Chinese male aged 19 years old underwent surgical resection of the lesions at the floor of the mouth and base of the tongue at Shanghai First People’s Hospital in February 2016. The pathological stage of the patient was classified as pT1N0M0, G1, Stage I (AJCC v8) (**Table 1**). Following the surgical procedure, the patient presented with an absence of the tongue and encountered significant challenges in both oral intake and verbal communication. To address these issues, the patient was provided with enteral nutrition support through a nasogastric tube. The patient reported no family history of malignant tumors. An ultrasound was performed on June 14, 2018, revealing a dense low-echo mass measuring 53 × 47 × 55 mm, originating from the base of the tongue and extending to the bottom of the mouth (**Figure 1A**). The mass exhibited clear boundaries and an irregular shape, with abundant blood flow signals (**Figure 1B**). A computed tomography (CT) scan (**Figures 2A, D, G, J**) was conducted on June 16, 2018, showing a laminar soft tissue density shadow in the region of the right mandibular angle. On contrast-enhanced imaging, this area demonstrated heterogeneous enhancement. On July 13, 2018, a pathological report was released by the Shanghai First People’s Hospital. On July 15, 2018, Fudan University Shanghai Cancer Center issued a consultation report shown in **Table 1**. The efficacy assessment resulted in progressive disease (PD) according to the Response Evaluation Criteria in Solid Tumors (RECIST) version 1.1, with a disease-free survival (DFS) of 28 months.

**Table 1:** Pathological results.

Time	Pathological specimen	Pathological results	ImmunohistochemiIcal results
July 13, 2018	Post-surgical tumor specimen	The initial pathological diagnosis from the referring hospital indicated a low-grade fibrous tumor located at the base of the tongue. The consultation conclusion suggested that the spindle cell lesion at the base of the tongue might correspond to a low-grade fibroblastic/myofibroblastic tumor, based on microscopic features and immunohistochemical data provided by the original institution. Due to unclear boundaries in the surrounding tissue, it is recommended to review sections from 2016 to assess whether there has been any recurrence of the lesion. Further clinical evaluation is advised.	
July 15, 2018	Post-surgical tumor specimen	Macroscopic examination identified three gray-white tissue samples from the base of the tongue, with dimensions of 1.2 × 0.6× 0.5 cm, 1.5 × 1 × 0.5 cm, and 1.6 × 1.5 × 0.7 cm, respectively. These samples exhibited gray-white cross-sections and moderate consistency. Diagnosis: spindle cell tumor at the base of the tongue. A supplementary report dated July 5, 2018, confirmed a low-grade fibrous tumor at the same location.	Immunohistochemistry results were as follows: CD31 (-), CD34 (+), CD68 (+/-), Desmin (-), F8 (-), Ki-67 (+ 2%), SMA (+), Vim (+), S-100 (-), PGP9.5 (-), beta-catenin (-), STAT-6 (-).
July 20, 2022	Tumor specimens surgically resected following the treatment of recurrent lesions	Following the comprehensive treatment of a malignant tumor located at the base of the tongue: (1). The resected specimens from the cranial-base/ maxillofacial region exhibit spindle-cell soft tissue proliferation with degenerative alterations, significant lymphocytic infiltration, edema in specific interstitial regions, mucoid degeneration, calcification, and ossification. The tumor measures 10 × 10 × 7.5 cm. Based on the patient's medical history and morphological evaluation, this is interpreted as a recurrence of a low-grade fibromatosis/ myofibromatosis lesion with post-treatment effects. The tumor has extended into the mandibular tissue, the entire tongue, the tongue base, the epiglottis, adjacent salivary gland tissue, striated muscle tissue, and cartilage tissue. (2). Examination of the right vagus nerve reveals nerve fiber tissue infiltrated by peripheral lymphocytes and plasma cells. (3). Two lymph nodes from the left neck and one lymph node from the right neck display signs of chronic inflammation.	SMA (-), Desmin (minimal weak positivity), CD34 (vascular positivity), S-100 (-), SOX10 (-), ALK (-), CK (-), EMA (-), Myogenin (-), NTRK (-), beta-Catenin (plasma positivity in a small fraction), Ki-67 (+, 5%), Her-2 (-), PD-L1 (22C3) (CPS: approximately 30).

From July 2018, the patient received first-line therapy using anti-vascular endothelial growth factor receptor (VEGFR) targeted therapy with apatinib mesylate tablets, which discontinued due to treatment-related adverse effects (TRAEs) in July 2019. On November 18, 2020, a CT scan was performed at Shanghai Ninth People’s Hospital, identifying a soft tissue mass approximately measuring 81 × 79 mm at the base of the mouth. This mass displayed unclear boundaries and significant growth compared to findings from May 8, 2019. The mass exhibited heterogeneous density with a CT value of 48 Hounsfield Units (HU) and multiple calcification foci. The lesion extended into the submandibular region with indistinct margins relative to both bilateral submandibular glands. Localized bone absorption and destruction were noted on the lingual aspect of both mandibles. On December 17, 2020, a tracheotomy was performed under local anesthesia. A magnetic resonance imaging (MRI) was conducted on January 6, 2021, indicating a large mass located in the base of the tongue and the floor of the mouth following surgery. Bilateral enlarged cervical lymph nodes were also observed, with high suspicion for metastasis. At this time point, the patient was in the clinical stage of rT3N1M0, G1, Stage IIIB (AJCC v8), with a progression-free survival 1 (PFS1) of 28 months.

The patient, aged 23 years, was admitted to the Department of Radiotherapy, Zhejiang Cancer Hospital, 3 weeks after bronchotomy due to pharyngeal obstruction caused by masses at the base of the tongue and floor of the mouth in January 2021. The laryngoscopy performed on January 7, 2021, revealed a new growth at the base of the tongue, showing rough and congested mucosa that bled easily upon biopsy. From January 14, 2021, to February 10, 2021, the patient underwent intensity-modulated RT for a malignant tumor located at the base of the tongue. The RT treatment plan entailed delivering a total dose of 5750 centigray (cGy) to the gross tumor volume (GTV), divided into 25 fractions. The doses received by organs-at-risk were presented in **Table 2**. During the course of RT, no significant reduction in tumor size was observed. After the 20^th^ fraction, RT was temporarily halted to administer levofloxacin as an anti-infective treatment due to an acute upper respiratory tract infection. A chest radiograph conducted on April 27, 2021, revealed the presence of a tracheotomy tube (**Figure 1C**). Following the discontinuation of RT, laboratory examinations performed on February 15, 2021, demonstrated the following results: hemoglobin concentration was 90.0 g/l (reference range: 130–175 g/l), neutrophil percentage was 0.85 (reference range: 0.40 - 0.75), high-sensitivity C-reactive protein levels were elevated to 104.1 mg/L (reference range: 0–5 mg/l), and albumin levels decreased to 29.0 g/l (reference range: 40–55 g/l). Additionally, a sputum culture conducted on the same date confirmed the presence of Pseudomonas aeruginosa. The minimum inhibitory concentration of levofloxacin against the isolated bacteria was determined to be 1 µg/ml on February 16, 2021. The abnormality in hemoglobin concentration was categorized as a TRAE: Grade 2 anemia, in accordance with the Common Terminology Criteria for Adverse Events (CTCAE) Version 5.0.

**Table 2:** Doses received by organs-at-risk.

No.	Organs-at-risk	Doses received by organs-at-risk
1	brainstem	Dmax 35.64 Gy
2	spinal cord	Dmax 27.8 Gy;
3	left lens	Dmax 1.27 Gy;
4	right lens	Dmax 1.65 Gy
5	left optic nerve	Dmax 23.2 Gy;
6	right optic nerve	Dmax 2.38 Gy;
7	optic chiasm	Dmax 1.94 Gy;
8	left temporal lobe	Dmax 3.08 Gy
9	ight temporal lobe	Dmax 4.5 Gy.

Thereafter, targeted therapy with anlotinib capsules, a VEGFR-tyrosine kinase inhibitor (TKI) (8 mg orally, once daily on days 1 to 14, repeated every 3 weeks as a treatment cycle), was initiated. A CT scan (**Figures 2B, H, E, K**) conducted on March 16, 2021, revealed an irregular soft tissue density mass measuring approximately 79 × 65 × 88 mm in the area of the right mandibular angle following surgery for a malignant tongue tumor. The mass was characterized by multiple mottled dense shadows and destructive changes within adjacent bone structures. Subsequently, the patient underwent 18 cycles of immunotherapy with toripalimab injection (240 mg), 3 weeks for each cycle, administered from May 26, 2021, to June 14, 2022. On March 13, 2022, CT imaging (**Figures 2C, F, I, L**) indicated a mass with a maximum diameter of 84 mm located in the mandibular region; the mass showed calcification and destruction of adjacent bone structures. The efficacy assessment resulted in stable disease (SD) with tumor shrinkage according to the modified RECIST 1.1 for immune-based therapeutics (iRECIST).

On July 8, 2022, the patient experienced acute oral bleeding, which necessitated an emergency procedure that included neck debridement and hemostasis at Zhejiang Cancer Hospital. The surgical intervention involved extensive resection of craniofacial and oral malignant tumors, bilateral cervical lymph node dissection, horizontal ramus resection of the mandible, partial laryngectomy, reconstruction of laryngeal function, resection of hypopharyngeal lesions, and repair using bilateral pectoralis major myocutaneous flaps. Postoperatively, the patient received anti-infection therapy and fluid replacement. The postoperative pathological report, dated July 20, 2022 (**Figure 1**) indicated significant increase in tumor calcification and ossification following treatment with anlotinib in combination with toripalimab, which may reflect tumor necrosis and could be associated with an improved prognosis. The oral bleeding was deemed a TRAE of anlotinib capsules. Based on the grading criteria of CTCAE v5.0, it was categorized as Grade 4, leading to the permanent cessation of anlotinib capsule use thereafter. From February 15, 2021, to July 25, 2022, spanning approximately one and a half years, platelet counts varied between 504 × 10^9^/l and 710 × 10^9^/l. By July 25, 2022, platelet levels had reduced to 357 × 10^9^/l, nearing the upper limit of the normal range, and subsequently remained within normal limits. From September 1, to November 30, 2022, the patient received intravenous infusions of toripalimab injection at a dosage of 240 mg for 5 cycles of immunotherapy. A CT scan was conducted on November 17, 2022, revealing postoperative changes indicative of a malignant tumor located at the base of the tongue. Additionally, scattered patchy and miliary shadows were observed in the right lung; some lesions were deemed inflammatory in nature. From August 25, to October 10, 2023, the patient received further intravenous infusions of toripalimab injection at a dose of 240 mg for continued 3 cycles of immunotherapy. Subsequently, the patient declined additional anti-tumor treatments and was provided with nasogastric feeding along with nutritional support. The nasogastric tubes were routinely replaced within our department. At present, the patient is undergoing nasogastric feeding therapy and has not experienced chills, fever, or intense pain. Through active monitoring and appropriate strategies, potential side effects related to the treatment can be effectively managed. According to the most recent follow-up until February 2025, the patient experienced a PFS2 of 48 months from initiation of RT, with an overall survival (OS) of 9 years.

## 3 Discussion

This report presents the case of a 19-year-old male with LGMS. LGMS is a rare STS of unknown etiology (20). Microscopically, all cases exhibit a neoplasm composed of oval to spindle-shaped cells within a fibrous stroma characterized by myxoid and dense areas. Atypical mitosis and prominent nucleoli are also observed (16). Histologically, the tumors are composed of slender spindle cells with eosinophilic cytoplasm, exhibiting fusiform, tapering, wavy, or plump ovoid shapes. The nuclei show a vesicular appearance and feature small central eosinophilic nucleoli (21). Ultrastructurally, the tumor cells displayed abundant rough endoplasmic reticulum and longitudinally arranged fine filaments with focal densities present in the cytoplasm (21). In a previous study, the immunohistochemical analysis revealed positivity for smooth muscle actin in 12 out of 13 cases, actin, muscle-specific (HHF35) in 2 out of 4 cases, β-catenin in 3 out of 5 cases, desmin in 3 out of 11 cases, and Ki-67 levels ranging from 5% to 50%. H-caldesmon was negative across all examined cases (16). In the other study, immunohistochemically, the tumor cells express smooth muscle actin (18/20), muscle-specific actin (16/20), fibronectin (20/20), and desmin (2/20) (21). In this case, compared to the initial post-surgical tumor specimens, the pathological features of recurrent samples exhibit soft tissue proliferation accompanied by degenerative changes, significant lymphocytic infiltration, edema in specific interstitial regions, mucoid degeneration, calcification, and ossification. Additionally, there is an increase in Ki-67 expression from 2% to 5%.

LGMS tends local recurrence, whereas metastasis remains relatively rare (22), as well as bony involvement (4). The average time to recurrence of LGMS was 19.2 months (18). To the contrary, there is a report indicating that LGMS is distinguished by its propensity for metastasis (8). The patient did not undergo RT or a combination of RT and chemotherapy in the postoperative period. Instead, he was administered a 12-month course of anti-VEGFR targeted therapy with apatinib mesylate tablets. This rare tumor presents challenges in diagnosis and lacks a standardized treatment protocol (23, 24). The treatment options for advanced STSs remain limited, with high rates of recurrence observed following the resection of localized disease (25). This patient experienced a recurrence 28 months post-surgery. The assessment and management of patients with STSs necessitate a multidisciplinary team with expertise in the treatment of these tumors (26). Surgical resection, aimed at obtaining an adequate negative margin, remains the primary treatment modality and is regarded as a definitive treatment for patients with localized low-grade tumors (27). The surgical margin obtained during the procedure is the most critical factor influencing local tumor control. Furthermore, the OS of the patient following resection primarily depends on the stage of the tumor (28). In addition, RT is a crucial element in the multidisciplinary management of STSs (29). Adjuvant RT, with or without chemotherapy, is recommended for patients diagnosed with high-grade STSs (27). However, Park et al. reported a case report, in which a microscopically precise resection was conducted, followed by postoperative RT for the third recurrent LGMS. This treatment resulted in both tumor recurrence and lung metastasis 7 months later (30). The impact of adjuvant chemotherapy on OS continues to be a subject of debate (31).

Novel combinations involving RT, chemotherapy, targeted therapy, vaccines, chimeric antigen receptor T-cell cells, and treatments that address other immune components of the tumor microenvironment are currently being explored to circumvent established resistance mechanisms (32). A retrospective cohort study assessed the efficacy of apatinib in combination with doxorubicin and ifosfamide (AI) as neoadjuvant therapy for high-risk STSs. The patients were divided into two treatment groups: the AI + apatinib group and the AI group. The regimen combining AI and ifosfamide demonstrated both safety and efficacy as a neoadjuvant therapy, achieving objective response rates of 53.85% compared to 29.17% (P = 0.047). Furthermore, the average change in target lesion size from baseline was significantly greater in the AI + apatinib group (-40.46 ± 40.30) than in the AI group (-16.31 ± 34.32) (P = 0.008). Additionally, the incidence of adverse effects related to neoadjuvant therapy and postoperative complications was comparable between the two groups (33).

Upon recurrence of the tumor, the patient was effectively treated with RT followed by anti-programmed death-1 (anti-PD-1) immunotherapy utilizing toripalimab injection combined with anti-VEGFR therapy using anlotinib capsules, achieving a response of SD with tumor shrinkage. Subsequently, surgical resection was performed to remove the residual lesions. However, it has been reported that the treatment options may be limited for patients with metastatic and recurrent sarcomas due to underlying organ dysfunction. Despite the implementation of these multimodal therapies, no significant improvement has been achieved in the prognosis of patients with sarcomas over the past decade (34).

VEGF and its receptors, known as VEGFRs, play a pivotal role in tumor angiogenesis and represent promising targets for anticancer therapies (35). Lin et al. presented a case report in which the patient with low-grade myofibroblastic sarcoma was effectively treated with apatinib mesylate tablets (250 mg/day) following the failure of imatinib, liposomal doxorubicin, and ifosfamide. The patient achieved a PFS of 8.5 months, with an OS of 17 months after initiating treatment with apatinib mesylate tablets. Notably, no grade 3 or 4 adverse effects were observed, apart from hand-foot syndrome (36). In comparison with the case report presented by Lin et al., the following similarities and differences in the findings are observed. In both reports, patients received apatinib treatment following recurrence of LGMS. The differences between the two case reports are outlined as follows. (a) The patient described in the case report by Lin et al. was a 45-year-old man (36), whereas this patient was 19 years of age at the time of initial diagnosis. (b) In the case reported by Lin et al., the patient presented with bilateral lung involvement, mediastinal lymph node enlargement, main pulmonary artery and branch embolism, and jejunal lesions at initial diagnosis, indicating advanced disease (36). In contrast, the lesion in this case involved the base of the tongue and the floor of the mouth at initial diagnosis, representing an early-stage condition. (c) In the case reported by Lin et al., the patient received first-line treatment with imatinib, followed by second-line chemotherapy consisting of liposomal doxorubicin and ifosfamide, and subsequently transitioned to apatinib as third-line therapy (36). In this patient, apatinib was administered as the first-line treatment. (d) In the case reported by Lin et al., the patient receiving apatinib treatment achieved a PFS of 8.5 months and an OS of 17 months (36), whereas the patient in the present case demonstrated a PFS of 28 months and an OS of 9 years.

In a multicenter, open-label, single-arm phase 2 study, anlotinib was used as a post-chemotherapy maintenance therapy and showed significant potential in terms of efficacy. Patients experienced a mPFS of 9.1 months, with an OS rate of 98.0%. The objective response rate (ORR) was recorded at 16%, and the disease control rate (DCR) reached 94%. Additionally, the treatment was associated with manageable toxicity levels among individuals with advanced STSs (37). The APROMISS study evaluated the efficacy and safety of dacarbazine versus anlotinib in patients with synovial sarcoma who had received two or more prior therapies. Anlotinib showed a longer PFS of 2.9 months compared to 1.6 months for dacarbazine. The incidence of adverse reactions with anlotinib was deemed tolerable (38). Anlotinib can inhibit the expression of programmed death-ligand 1 (PD-L1) (39).

PD-L1 expression may serve as a valuable prognostic indicator of unfavorable outcomes in patients diagnosed with STSs (40, 41). PD-L1 binds to its receptor, programmed death-1 (PD-1), activating the immune checkpoint response in T cells, which allows tumor cells to evade immune surveillance and resist conventional chemotherapy (42). As a result, this phenomenon has garnered significant attention in recent years (42). Immunohistochemical analysis revealed a PD-L1 expression with a combined positive score (CPS) of approximately 30 in this patient. The expression of PD-L1 in sarcomas ranges from 1.4% to 59%, with an average of 24% (43). This expression appears to be specific to the type of sarcoma (43). PD-L1 protein expresses significantly in cell lines of pleomorphic rhabdomyosarcoma, fibrosarcoma, and dedifferentiated liposarcoma (44). Notably, the majority of sarcomas do not express PD-L1 (43). Furthermore, elevated PD-L1 expression was correlated with reduced OS and diminished event-free survival in patients diagnosed with STSs (40). Immunotherapy has transformed the landscape of cancer treatment; however, many immunotherapy agents have not yet been approved for use in patients with STSs (45). An investigator-initiated, multicenter, single-group, phase 2 study was conducted to evaluate the efficacy of the anti-PD-L1 agent atezolizumab in both adult and pediatric patients with advanced alveolar soft part sarcoma (ASPS). The results indicated that atezolizumab was effective in inducing sustained responses in approximately one-third of patients suffering from advanced ASPS (46). In a single-center phase 2 trial, the combination of durvalumab and tremelimumab exhibited efficacy as an active treatment regimen for advanced or metastatic sarcoma (47). A pooled analysis of clinical trials investigating PD-1 or PD-L1 antagonists in patients with advanced STSs was conducted. This cohort comprised 39.8% of participants treated with anti-PD-1/PD-L1 as a monotherapy, achieving an ORR and non-progression rate (NPR) of 15.1% and 58.5%, respectively. Conversely, for patients receiving combination regimens, the ORR and NPR were recorded at 13.4% and 55.8%, respectively. Patients diagnosed with alveolar soft part sarcoma and undifferentiated pleomorphic sarcoma exhibited the highest response rates, while those with leiomyosarcoma demonstrated the lowest response rates. A low expression rate of PD-L1 was found, which was inconsistently correlated with objective responses. In conclusion, monotherapy of PD-1/PD-L1 antagonists shows limited efficacy in unselected cases of STSs (48).

Compared to tissue-based assessments of PD-L1, exosomal PD-L1 presents distinct advantages in terms of accessibility and its ability to reflect the tumor immune status dynamically. Nevertheless, challenges persist concerning the standardization of detection methods and the clinical interpretation of results (49). The levels of circulating exosomal PD-L1 were found to significantly elevate in patients with chronic hepatitis B and hepatocellular carcinoma as compared to healthy controls (50). A laboratory-based experimental study was conducted to simultaneously detect the levels of circulating tumor cells (CTCs) in PD-L1-positive patients suffering from hypopharyngeal and laryngeal cancers. The consistency of PD-L1 expression between CTCs and tissue specimens demonstrated a substantial agreement exceeding 70% (51). It has reported that the addition of pembrolizumab to docetaxel and cisplatin (TP) *ex vivo* leads to a greater suppression of monocyte chemoattractant protein 1 (MCP-1), interferon-γ (IFN-γ), and interleukin-6 production in head and neck squamous cell carcinoma than what is observed with TP alone. This enhancement is associated with improved survival outcomes (52). Furthermore, the inhibition of PD-L1 resulted in a significant increase in tumor necrosis factor-α (TNF-α) levels, accompanied by a concurrent decrease in interleukin-10 levels (51).

In the case of this LGMS patient with two relapse, palliative RT, followed by a combination of immunotherapy and anti-VEGFR targeted therapy, resulted in a significant increase in calcification and ossification within both the tumor bed and the lymph nodes located in the drainage area. LGMS is characterized by its invasive nature and the presence of calcification. It has reported that significant calcification and ossification were observed in 2 out of 14 cases (16). The 5-year OS rate of 82.6% for extensively calcified synovial sarcoma is significantly higher than the reported 5-year OS rates for synovial sarcoma, which range from 25% to 51% (53). This finding indicates that tumor tissue calcifications are independently and substantially associated with prolonged OS in patients diagnosed with synovial sarcomas (54). Calcification and ossification are imaging characteristics commonly observed in STSs. A univariate analysis of 20 pathologically confirmed cases of primary dedifferentiated liposarcoma in the retroperitoneum revealed that calcification or ossification significantly impacted overall survival (55). Notably, the occurrence of calcification or ossification is more commonly associated with well-differentiated liposarcoma; however, it has also been reported as infrequent in other malignant soft tissue lesions (56). Highly cellular lesions exhibited poor levels of calcification and ossification, whereas heavily calcified lesions typically demonstrated relatively low cellularity in patients diagnosed with primary synovial chondromatosis (57). Patients with dedifferentiated liposarcoma exhibiting osteogenic differentiation showed a tendency for earlier local recurrences; nevertheless, this did not consistently lead to adverse life outcomes (58).

For patients with STSs, several relevant studies have indicated that immunotherapy following palliative RT may produce a synergistic therapeutic effect, offering valuable clinical insights. PD-L1 expression on human STSs and tumor-associated macrophages (TAM) appears to increase following preoperative RT in a cohort of 46 patients with Stage II-III STSs. These patients received preoperative RT at doses (50-50.4 Gy in 25–28 fractions) prior to surgical resection (59). Complete surgical resection remains the most critical treatment strategy for undifferentiated pleomorphic sarcoma (UPS), while adjuvant RT or chemotherapy has proven inadequate in enhancing survival rates. Immunotherapy may represent a significant advancement in the management of UPS patients (60). RT further enhances the release of tumor-associated antigens, activates antigen-presenting cells and dendritic cells, increases cytokine production, modifies the tumor microenvironment, and stimulates the body’s immune system to mount antitumor immune responses. Such modifications to the tumor microenvironment induced by RT can significantly amplify the body’s antitumor immune effects (61),

Why was the combination of immunotherapy and anti-angiogenic therapy chosen for the management of this patient? (a). The impact of the VEGFR pathway on the immune microenvironment, particularly regarding PD-L1 expression in sarcoma, is presented as follows. Apatinib inhibits migration and invasion as well as PD-L1 expression in osteosarcoma by targeting signal transducer and activator of transcription 3 (STAT3) (62). In another experimental study, apatinib inhibits the expression of PD-L1 by targeting the VEGFR2/STAT3 signaling pathway in lung cancer (63). Sunitinib, a VEGFR-TKI, inhibits PD-L1 expression in osteosarcoma by targeting STAT3 and remodels the immune system in tumor-bearing mice (64). PD-L1 expression may elevate in a subset of metastatic clear cell renal cell carcinoma patients who are resistant to VEGFR-TKI, potentially through the mammalian target of rapamycin pathway (65). Lenvatinib, a VEGFR-TKI, reduced the population of tumor-associated macrophages while simultaneously increasing the presence of plasmacytoid dendritic cells during the early stages of treatment (66). The immune escape mechanism in laryngeal carcinoma is promoted by the activation of VEGFR1/transforming growth factor-β signaling, which facilitates PD-L1 expression in M2-like TAMs (67). (b). On the other hand, the expression of PD-L1 also influences VEGFR levels. PD-L1 modulates angiogenesis by engaging in the c-JUN/VEGFR2 signaling axis in ovarian cancer (68). Elevated preoperative serum levels of PD-L1 are indicative of a poor response to VEGF-targeted therapy and suggest an unfavorable prognosis in renal cell carcinoma (69). (c). The blockade of angiogenesis can modulate the tumor microenvironment and enhance the efficacy of concurrent immunotherapies. Monotherapies targeting VEGFR-2 and PD-L1 elicited both unique and overlapping patterns of immune gene expression, while combination therapy resulted in a significantly enhanced immune activation signature (70). Sun et al. reported the case of a recurrent patient diagnosed with LGMS of the pharynx who received a treatment regimen combining anlotinib and pembrolizumab for 4 cycles, resulting in a partial response (24). In another case report, the combination of anlotinib and pembrolizumab exhibited a relatively favorable response in patients with LGMS of the pancreas (71). A single-center, observational, prospective study showed that the combination of anlotinib and anti-PD-1 antibodies demonstrated promising and durable antitumor efficacy with an acceptable toxicity profile in patients with advanced tumors. Additionally, this treatment regimen linked to favorable changes in serum levels of interleukin-2, interleukin-4, interleukin-10, TNF-α, and IFN-γ, as well as alterations in circulating immune cell subsets among clinical responders (72).

In comparison with the case reported by Sun et al., the following similarities and differences are observed. The similarity between the two case reports lies in the use of a combination therapy involving an anti-PD-1 antibody and anlotinib capsules for the treatment of recurrent LGMS, as well as discontinuation of anlotinib capsules due to adverse drug reactions. The differences are manifested in the following aspects. (a) Treatment with immune checkpoint inhibitors in conjunction with anlotinib capsules was initiated after the second relapse in the present case, whereas it was administered following the first relapse in the case reported by Sun et al. (24). (b) Anti-PD-1 inhibitors were administered in both this case and Sun et al.’s study, with toripalimab utilized in the present case and pembrolizumab employed in the latter (24). (c) In this instance, the patient underwent palliative radiotherapy prior to treatment with an anti-PD-1 inhibitor and anlotinib, whereas in the case reported by Sun et al., radiotherapy was not administered (24). (d) In the present case, the patient underwent palliative radiotherapy followed by immunotherapy in combination with anlotinib, after which significant calcification and ossification were observed in the lesion site and draining lymph nodes postoperatively. In contrast, the case reported by Sun et al. did not document similar pathological changes (24). (e) With regard to efficacy assessment, our patient achieved stable disease with tumor shrinkage, whereas in the case reported by Sun et al., the treatment response was classified as complete response (24).

The dilemmas encountered by the patient before undergoing combined immunotherapy and anti-angiogenic targeted therapy following RT include: (a) Following surgical removal of LGMS located at the base of the tongue, the patient was unable to perform swallowing exercises and relied on a nasogastric tube for nutritional intake. Concurrently, the patient experienced a loss of speech function and exhibited reluctance in engaging with the external environment. (b) He underwent tracheostomy and fitted with a tracheal cannula for the purpose of ventilatory support. (c) The patient developed a post-RT infection and required treatment with levofloxacin injection as part of the anti-infective regimen.

For this patient, the following factors potentially contributed to the favorable therapeutic outcome. (a) The patient is a young individual with LGMS and a PD-L1 CPS of about 30. The expression of PD-L1 in various STSs among the young population has confirmed, revealing its independent negative prognostic significance (73). However, this finding suggests that the PD-1/PD-L1 axis may serve as a potential therapeutic target for treating young patients with STSs (73). (b) The patient underwent RT before receiving immunotherapy and anti-angiogenic targeted therapy. An increased expression of PD-L1 was observed in human STS tumors and tumor-associated macrophages following RT (59). (c) Anti-angiogenic targeted therapy with anlotinib may enhance the clinical efficacy of toripalimab-based immunotherapy. (d) The patient was advised to undergo regular follow-up examinations to facilitate the early detection of recurrence or metastasis (7).

However, in the treatment of this patient, we contend that several key factors influence the outcomes of patient treatment, as outlined below. (a) Apatinib mesylate tablets were interrupted due to TRAE at the stage of postoperative adjuvant therapy, so it is necessary to select appropriate anti-vascular targeted therapy drugs or other therapeutic measures to prolong DFS of patients. (b) In the second-line treatment phase following the patient’s recurrence, the planned RT dosages were not completed as scheduled and were stopped after the 20^th^ session. This interruption could result in an inadequate radiation doses, potentially impacting the effectiveness of the treatment. (c) At the time of relapses, a biopsy was not performed on the lesions or the suspicious lymph nodes. (d) PD-L1 expression was identified exclusively in the surgical specimens of the treated residual tumor following the second tumor recurrence, but not in the specimens obtained during the initial surgical intervention.

Regarding the patient’s outlook on the future, he was unable to communicate verbally due to the surgical excision of the tongue base. The level of communication between the patient and healthcare providers during his hospitalization was relatively limited. Following discussions with the patient’s parents, they indicated that the patient’s positive expectations for the future had improved compared to the period immediately following the palliative surgery.

Based on this case report, we propose the hypothesis that RT followed by treatment with toripalimab in combination with anlotinib may yield an acceptable therapeutic effect for relapsed LGMS, accompanied by manageable TRAEs. Nevertheless, this hypothesis requires further validation through higher-level evidence-based medical research.

## Data Availability

The original contributions presented in the study are included in the article/supplementary material. Further inquiries can be directed to the corresponding author.
